# Supplementary material for: Association between inflammatory biomarkers and cognitive aging
Source: PLoS One. 2022 Sep 9;17(9):e0274350. doi: 10.1371/journal.pone.0274350 (PMC9462682; doi:10.1371/journal.pone.0274350)
Supplement: S1 Table — a). Lower and upper detectable limits and the upper CV limit of the protein biomarkers included. Values outside the detectable limits were set to the lower or upper detectable limits respectively and were set to unknown if the CV was above the threshold. a a. Details on protein biomarker measurement and quality control have been described in Ho JE, Lyass A, Courchesne P, Chen G, Liu C, Yin X, et al. Protein Biomarkers of Cardiovascular Disease and Mortality in the Community. Journal of the American Heart Association: Cardiovascular and Cerebrovascular Disease. 2018 Jul 1;7(14). doi:10.1161/JAHA.117.008108. b). Number of cases where the protein biomarkers are below, within, or above the detectable limits, or are set to missing because CV was above the upper limit threshold or are missing. a a. Sample sizes for all biomarkers are different due to the different number of missingness. b. Not all individuals were included for all protein assays. (PDF) [file pone.0274350.s001.pdf]

S1 Table a). Lower and upper detectable limits and the upper CV limit of the protein biomarkers included. Values outside the detectable limits were set to the lower or upper detectable limits respectively and were set to unknown if the CV was above the threshold. [a]

Biomarker	Lower detectable limit (pg/mL)	Upper detectable limit (pg/mL)	Upper CV limit
**CD14**	58200	239000000	55.8
**CD163**	1650	1180000	21.8
**CD5L**	2240	9540000	17.7
**CD56**	7040	6790000	10.3
**CD40L**	19.4	46100	61.5
**CXCL16**	73.8	17100	42.1
**SDF1**	59.4	197000	46.1
**DPP4**	2470	1800000	23.5
**sGP130**	417	--	30.2
**sRAGE**	103	75300	38.7
**MPO**	102	--	48.1

a. Details on protein biomarker measurement and quality control have been described in Ho JE, Lyass A, Courchesne P, Chen G, Liu C, Yin X, et al. Protein Biomarkers of Cardiovascular Disease and Mortality in the Community. *Journal of the American Heart Association: Cardiovascular and Cerebrovascular Disease*. 2018 Jul 1;7(14). doi:10.1161/JAHA.117.008108

S1 Table b). Number of cases where the protein biomarkers are below, within, or above the detectable limits, or are set to missing because CV was above the upper limit threshold or are missing. [a]

	Neuropsychological Test Sample (n = 2358)										
	**CD14**	**CD163**	**CD5L**	**CD56**	**CD40L**	**CXCL16**	**SDF1**	**DPP4**	**sGP130**	**sRAGE**	**MPO**
Below measurable range	0	0	0	0	372	0	8	0	2	0	2
Within measurable range	2,389	2,380	2,384	2,383	2,015	2,389	2,379	2,382	2,390	2,388	2,390
Above measurable range	0	0	0	0	0	0	0	0	0	0	0
Set to missing because CV was above allowable limit	1	6	7	7	7	5	5	6	2	6	2
Missing data [b]	4	8	3	4	0	0	2	6	0	0	0
	**MRI Sample (n = 2100)**										
	**CD14**	**CD163**	**CD5L**	**CD56**	**CD40L**	**CXCL16**	**SDF1**	**DPP4**	**sGP130**	**sRAGE**	**MPO**
Below measurable range	0	0	0	0	333	0	7	0	2	0	2
Within measurable range	2,130	2,121	2,126	2,124	1,796	2,130	2,121	2,124	2,131	2,129	2,131
Above measurable range	0	0	0	0	0	0	0	0	0	0	0
Set to missing because CV was above allowable limit	1	6	6	7	6	5	5	5	2	6	2
Missing data	4	8	3	4	0	0	2	6	0	0	0
	**Dementia Sample (n = 1616)**										
	**CD14**	**CD163**	**CD5L**	**CD56**	**CD40L**	**CXCL16**	**SDF1**	**DPP4**	**sGP130**	**sRAGE**	**MPO**
Below measurable range	0	0	0	0	253	0	6	0	1	0	1
Within measurable range	1,649	1,637	1,647	1,647	1,393	1,648	1,636	1,637	1,649	1,642	1,649
Above measurable range	0	0	0	0	0	0	0	0	0	0	0
Set to missing because CV was above allowable limit	0	7	2	2	4	2	7	7	0	8	0
Missing data	1	6	1	1	0	0	1	6	0	0	0

a. Sample sizes for all biomarkers are different due to the different number of missingness.
b. Not all individuals were included for all protein assays.
