# Supplementary material for: Association between inflammatory biomarkers and cognitive aging
Source: PLoS One. 2022 Sep 9;17(9):e0274350. doi: 10.1371/journal.pone.0274350 (PMC9462682; doi:10.1371/journal.pone.0274350)
Supplement: S2 Table — a). Distribution of participants protein biomarkers in the dementia, neuropsychological test, and MRI study samples. b). Participants’ neuropsychological testing scores and brain MRI measures stratified by APOE ε4 carrier status. (PDF) [file pone.0274350.s002.pdf]

S2 Table a). Distribution of participants protein biomarkers in the dementia, neuropsychological test, and MRI study samples.

Biomarker	Neuropsychological Test Sample n=2358	MRI Sample n=2100	Dementia Sample n=1616
**CD14, pg/mL, median (Q1, Q3)**	1.65e+07 (1.44e+07, 1.90e+07)	1.58e+07 (1.38e+07, 1.83e+07)	1.58e+07 (1.38e+07, 1.83e+07)
**CD163, pg/mL, median (Q1, Q3)**	1.17e+05 (8.32e+04, 1.56e+05)	1.09e+05 (7.68e+04, 1.47e+05)	1.08e+05 (7.56e+04, 1.48e+05)
**CD5L, pg/mL, median (Q1, Q3)**	2.35e+05 (1.81e+05, 3.09e+05)	2.20e+05 (1.69e+05, 2.85e+05)	2.20e+05 (1.69e+05, 2.85e+05)
**CD56, pg/mL, median (Q1, Q3)**	2.93e+05 (2.44e+05, 3.46e+05)	2.84e+05 (2.38e+05, 3.37e+05)	2.83e+05 (2.38e+05, 3.35e+05)
**CD40L, pg/mL, median (Q1, Q3)**	3.35e+01 (2.38e+01, 4.94e+01)	3.27e+01 (2.34e+01, 4.67e+01)	3.27e+01 (2.34e+01, 4.66e+01)
**CXCL16, pg/mL, median (Q1, Q3)**	1.16e+03 (9.78e+02, 1.35e+03)	1.12e+03 (9.49e+02, 1.31e+03)	1.12e+03 (9.49e+02, 1.31e+03)
**SDF1, pg/mL, median (Q1, Q3)**	3.41e+02 (2.70e+02, 4.41e+02)	3.40e+02 (2.68e+02, 4.46e+02)	3.41e+02 (2.68e+02, 4.48e+02)
**DPP4, pg/mL, median (Q1, Q3)**	5.56e+04 (4.21e+04, 7.11e+04)	5.66e+04 (4.32e+04, 7.37e+04)	5.63e+04 (4.30e+04, 7.39e+04)
**sGP130, pg/mL, median (Q1, Q3)**	6.22e+04 (5.56e+04, 6.93e+04)	6.06e+04 (5.41e+04, 6.77e+04)	6.05e+04 (5.40e+04, 6.75e+04)
**sRAGE, pg/mL, median (Q1, Q3)**	3.30e+03 (2.64e+03, 4.15e+03)	3.39e+03 (2.72e+03, 4.20e+03)	3.40e+03 (2.73e+03, 4.20e+03)
**MPO, pg/mL, median (Q1, Q3)**	9.84e+03 (7.63e+03, 1.26e+04)	9.47e+03 (7.39e+03, 1.20e+04)	9.41e+03 (7.33e+03, 1.20e+04)

S2 Table b). Participants' neuropsychological testing scores and brain MRI measures stratified by *APOE* ε4 carrier status.

Neuropsychological Test Sample		
**Neuropsychological tests**	***APOE* ε4 carriers (n = 522)**	**Non-carriers (n = 1790)**
**LMD, mean (SD)**	10.3 (3.9)	10.5 (3.6)
**PASD, mean (SD)**	8.1 (3.4)	8.1 (3.4)
**VRD, mean (SD)**	16.9 (3.6)	16.7 (3.6)
**TRAILSBA, mean (SD)**	1.0 (1.2)	0.9 (0.9)
**SIM, mean (SD)**	8.3 (1.6)	8.2 (1.5)
**HVOT, mean (SD)**	25.2 (3.1)	25.0 (3.1)
**BNT30, mean (SD)**	27.4 (2.7)	27.3 (2.8)

MRI Sample		
**brain MRI measures**	***APOE* ε4 carriers (n = 472)**	**Non-carriers (n = 1586)**
**TCBV, cm^3, mean (SD)**	957.8 (99.0)	954.6 (101.6)
**HPV, cm^3, mean (SD)**	6.6 (0.7)	6.6 (0.7)
**WMH, cm^3, mean (SD)**	1.2 (2.3)	1.3 (3.2)
**CGV, cm^3, mean (SD)**	498.5 (49.1)	497.4 (49.4)
**FGV, cm^3, mean (SD)**	175.8 (18.4)	175.6 (18.3)
**OGV, cm^3, mean (SD)**	60.3 (8.2)	59.9 (8.1)
**PGV, cm^3, mean (SD)**	97.5 (10.3)	97.2 (10.3)
**TGV, cm^3, mean (SD)**	123.7 (12.9)	123.5 (13.2)
**TCBV, cm^3, mean (SD)**	606.5 (57.5)	605.4 (58.1)
