# Supplementary material for: Association between inflammatory biomarkers and cognitive aging
Source: PLoS One. 2022 Sep 9;17(9):e0274350. doi: 10.1371/journal.pone.0274350 (PMC9462682; doi:10.1371/journal.pone.0274350)
Supplement: S3 Table — Both protein biomarkers and neuropsychological test scores are rank normalized to mean 0 and standard deviation 1. a. Model 1 covariates (age, sex, education level, time distance between exam 7 and the Neuropsychological testing, and retest indicator) were included. b. FDR ≤ 0.1 threshold to account for multiple testing. (PDF) [file pone.0274350.s003.pdf]

S3 Table. Cross-sectional association of protein biomarkers with neuropsychological test performance using linear mixed effect models adjusting for Model 1 covariates [a]. Both protein biomarkers and neuropsychological test scores are rank normalized to mean 0 and standard deviation 1.

	LMD		VRD		SIM		TRAILSBA		PASD		HVOT		BNT30	
**Biomarker**	**Effect ± SE**	**FDR [b]**	**Effect ± SE**	**FDR**	**Effect ± SE**	**FDR**	**Effect ± SE**	**FDR**	**Effect ± SE**	**FDR**	**Effect ± SE**	**FDR**	**Effect ± SE**	**FDR**
**CD14**	0.0015 ± 0.02	1.00	-0.045 ± 0.02	0.16	**-0.060 ± 0.02**	**0.041**	0.017 ± 0.02	0.78	-0.027 ± 0.02	0.54	-0.034 ± 0.02	0.38	-0.0051 ± 0.02	0.97
**CD163**	-0.020 ± 0.02	0.76	-0.034 ± 0.02	0.37	-0.045 ± 0.02	0.15	0.028 ± 0.02	0.59	0.0043 ± 0.02	0.97	-0.034 ± 0.02	0.37	-0.044 ± 0.02	0.15
**CD5L**	0.038 ± 0.02	0.30	-0.0064 ± 0.02	0.96	-0.023 ± 0.02	0.61	0.0018 ± 0.02	1.00	0.010 ± 0.02	0.89	-0.019 ± 0.02	0.76	-0.0010 ± 0.02	1.00
**CD56**	-0.040 ± 0.02	0.35	0.0062 ± 0.02	0.97	0.015 ± 0.02	0.81	-0.00011 ± 0.02	1.00	0.0042 ± 0.02	0.97	-0.025 ± 0.02	0.66	-0.017 ± 0.02	0.78
**CD40L**	0.019 ± 0.02	0.78	-0.0069 ± 0.02	0.96	**-0.070 ± 0.02**	**0.025**	-0.0070 ± 0.02	0.96	-0.0073 ± 0.02	0.96	-0.031 ± 0.02	0.54	-0.025 ± 0.02	0.61
**CXCL16**	0.019 ± 0.02	0.76	0.0061 ± 0.02	0.96	-0.020 ± 0.02	0.76	0.048 ± 0.02	0.15	-0.0045 ± 0.02	0.97	-0.016 ± 0.02	0.80	-0.0037 ± 0.02	0.97
**SDF1**	0.014 ± 0.02	0.85	-0.0099 ± 0.02	0.94	-0.022 ± 0.02	0.70	-0.012 ± 0.02	0.89	0.00080 ± 0.02	1.00	0.00088 ± 0.02	1.00	0.013 ± 0.02	0.84
**DPP4**	0.0078 ± 0.02	0.96	0.017 ± 0.02	0.76	0.0033 ± 0.02	0.98	0.013 ± 0.02	0.85	0.0079 ± 0.02	0.95	-0.00021 ± 0.02	1.00	0.00097 ± 0.02	1.00
**sGP130**	-0.020 ± 0.02	0.76	0.0015 ± 0.02	1.00	-0.043 ± 0.02	0.19	0.010 ± 0.02	0.94	0.0093 ± 0.02	0.94	-0.018 ± 0.02	0.78	-0.0078 ± 0.02	0.96
**sRAGE**	-0.020 ± 0.02	0.76	0.045 ± 0.02	0.15	**0.050 ± 0.02**	**0.097**	-0.025 ± 0.02	0.61	0.034 ± 0.02	0.30	-0.00058 ± 0.02	1.00	0.014 ± 0.02	0.81
**MPO**	0.015 ± 0.02	0.81	-0.032 ± 0.02	0.37	**-0.061 ± 0.02**	**0.025**	-0.0089 ± 0.02	0.95	0.0044 ± 0.02	0.97	-0.030 ± 0.02	0.47	-0.024 ± 0.02	0.59

a. Model 1 covariates (age, sex, education level, time distance between exam 7 and the Neuropsychological testing, and retest indicator) were included.
b. $\mathrm{FDR} \leq 0.1$ threshold to account for multiple testing.
