# Supplementary material for: Association between inflammatory biomarkers and cognitive aging
Source: PLoS One. 2022 Sep 9;17(9):e0274350. doi: 10.1371/journal.pone.0274350 (PMC9462682; doi:10.1371/journal.pone.0274350)
Supplement: S4 Table — Protein biomarkers are rank normalized to mean 0 and standard deviation 1. a. Model 1 covariates (age, age2, sex, age-sex interaction, time distance between exam 7 and the brain MRI scan) were included. Total and regional brain volumes and WMH volume were as percentage of total cranial volume, WMH was also log transformed. b. FDR ≤ 0.1 threshold to account for multiple testing. (PDF) [file pone.0274350.s004.pdf]

S4 Table. Cross-sectional association of protein biomarkers with brain MRI measures using linear mixed effect models adjusting for Model 1 covariates [a]. Protein biomarkers are rank normalized to mean 0 and standard deviation 1.

	**TCBV**		**HPV**		**WMH**		**CGV**		**FGV**		**OGV**		**PGV**		**TGV**	
**Biomarker**	**Effect ± SE**	**FDR [b]**	**Effect ± SE**	**FDR**	**Effect ± SE**	**FDR**	**Effect ± SE**	**FDR**	**Effect ± SE**	**FDR**	**Effect ± SE**	**FDR**	**Effect ± SE**	**FDR**	**Effect ± SE**	**FDR**
**CD14**	**-0.14 ± 0.04**	**0.068**	-0.0022 ± 0.00	0.23	0.034 ± 0.02	0.43	-0.073 ± 0.04	0.27	-0.013 ± 0.02	0.78	-0.015 ± 0.01	0.46	-0.018 ± 0.01	0.43	-0.031 ± 0.01	0.14
**CD163**	0.0020 ± 0.04	0.99	0.00036 ± 0.00	0.94	-0.0017 ± 0.02	0.99	0.019 ± 0.04	0.86	0.011 ± 0.02	0.83	0.0082 ± 0.01	0.78	-0.0031 ± 0.01	0.98	0.0023 ± 0.01	0.99
**CD5L**	**-0.13 ± 0.04**	**0.068**	-0.0022 ± 0.00	0.20	0.0030 ± 0.02	0.99	0.057 ± 0.03	0.43	0.019 ± 0.02	0.61	0.0063 ± 0.01	0.84	0.0065 ± 0.01	0.84	0.015 ± 0.01	0.63
**CD56**	0.13 ± 0.05	0.12	0.0014 ± 0.00	0.60	0.0035 ± 0.02	0.99	0.0081 ± 0.04	0.99	0.012 ± 0.02	0.84	-0.0074 ± 0.01	0.83	0.012 ± 0.01	0.72	-0.0019 ± 0.01	0.99
**CD40L**	-0.0043 ± 0.05	0.99	-0.0013 ± 0.00	0.65	-0.030 ± 0.02	0.54	0.050 ± 0.04	0.61	0.0080 ± 0.02	0.87	0.017 ± 0.01	0.43	0.0074 ± 0.01	0.84	0.024 ± 0.01	0.37
**CXCL16**	-0.12 ± 0.04	0.12	-0.00057 ± 0.00	0.85	-0.048 ± 0.02	0.14	-0.036 ± 0.03	0.72	-0.0082 ± 0.02	0.86	-0.0089 ± 0.01	0.76	-0.0028 ± 0.01	0.99	-0.012 ± 0.01	0.72
**SDF1**	0.039 ± 0.04	0.78	0.00022 ± 0.00	0.99	-0.023 ± 0.02	0.66	0.012 ± 0.04	0.95	-0.015 ± 0.02	0.76	0.0014 ± 0.01	0.99	0.0096 ± 0.01	0.78	0.013 ± 0.01	0.72
**DPP4**	0.058 ± 0.04	0.59	-0.00044 ± 0.00	0.87	-0.046 ± 0.02	0.14	-0.0014 ± 0.03	0.99	-0.0037 ± 0.01	0.99	-0.0058 ± 0.01	0.84	0.0046 ± 0.01	0.88	0.012 ± 0.01	0.72
**sGP130**	-0.038 ± 0.05	0.78	-0.00012 ± 0.00	0.99	-0.018 ± 0.02	0.78	0.0040 ± 0.04	0.99	-0.000066 ± 0.02	1.00	0.0075 ± 0.01	0.82	0.0091 ± 0.01	0.78	-0.016 ± 0.01	0.61
**sRAGE**	**0.19 ± 0.04**	**0.0012**	0.0019 ± 0.00	0.27	-0.022 ± 0.02	0.71	0.094 ± 0.04	0.12	0.033 ± 0.02	0.23	0.019 ± 0.01	0.27	0.017 ± 0.01	0.43	0.019 ± 0.01	0.44
**MPO**	0.019 ± 0.04	0.88	0.00048 ± 0.00	0.87	0.012 ± 0.02	0.84	0.0066 ± 0.04	0.99	-0.0084 ± 0.02	0.86	-0.000035 ± 0.01	1.00	-0.00051 ± 0.01	0.99	0.0069 ± 0.01	0.86

a. Model 1 covariates (age, age2, sex, age-sex interaction, time distance between exam 7 and the brain MRI scan) were included. Total and regional brain volumes and WMH volume were as percentage of total cranial volume, WMH was also log transformed.

b. $FDR \leq 0.1$ threshold to account for multiple testing.
