# Supplementary material for: Association between inflammatory biomarkers and cognitive aging
Source: PLoS One. 2022 Sep 9;17(9):e0274350. doi: 10.1371/journal.pone.0274350 (PMC9462682; doi:10.1371/journal.pone.0274350)
Supplement: S5 Table — a. Model 2 covariates includes Model 1 covariates with additional adjustment for APOE ε4 carrier status and CVD risk factors (SBP, treatment for hypertension, BMI, current smoking status, total cholesterol levels, HDL, presence of diabetes, prevalent AF, and prevalent CVD). b. FDR ≤ 0.1 threshold to account for multiple testing. (PDF) [file pone.0274350.s005.pdf]

S5 Table. Sensitivity analyses results: association between protein biomarkers and incident all-cause dementia within 10 years follow-up using cox proportional hazard regression models adjusting for Model 2 covariates [a].

Biomarker	Hazard Ratio	95% CI	p-value	FDR [b]
**CD14**	1.17	(0.97, 1.41)	0.11	0.39
**CD163**	1.07	(0.88, 1.30)	0.48	0.65
**CD5L**	1.13	(0.97, 1.32)	0.12	0.39
**CD56**	1.03	(0.85, 1.26)	0.74	0.74
**CD40L**	1.15	(0.95, 1.39)	0.14	0.39
**CXCL16**	1.06	(0.88, 1.27)	0.53	0.65
**SDF1**	1.17	(0.97, 1.41)	0.10	0.39
**DPP4**	0.92	(0.77, 1.09)	0.32	0.5
**sGP130**	1.09	(0.92, 1.30)	0.32	0.5
**sRAGE**	0.89	(0.71, 1.10)	0.28	0.5
**MPO**	0.97	(0.81, 1.15)	0.69	0.74

a. Model 2 covariates includes Model 1 covariates with additional adjustment for *APOE* ε4 carrier status and CVD risk factors (SBP, treatment for hypertension, BMI, current smoking status, total cholesterol levels, HDL, presence of diabetes, prevalent AF, and prevalent CVD).
b. $\mathrm{FDR} \leq 0.1$ threshold to account for multiple testing.
