# Supplementary figures and images for: Association between inflammatory biomarkers and cognitive aging
Source: PLoS One. 2022 Sep 9;17(9):e0274350. doi: 10.1371/journal.pone.0274350 (PMC9462682; doi:10.1371/journal.pone.0274350)

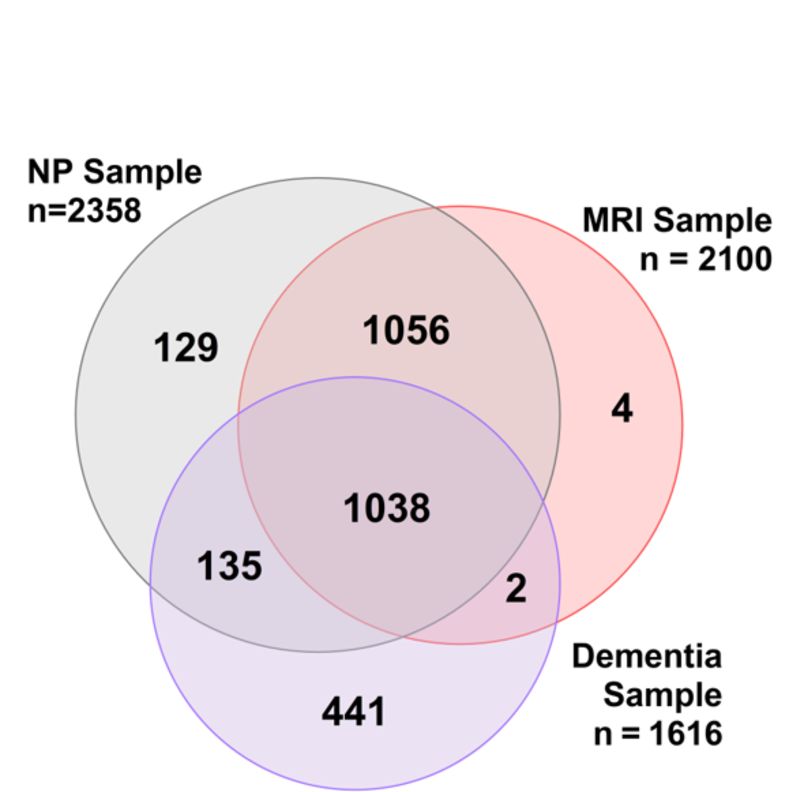

Supplement: S1 Fig — Numbers labeled are number of participants included in each scenario. There are 1038 participants included in all three subsamples; 2094 in both neuropsychological test and MRI subsample; 1171 in both neuropsychological test and dementia subsample; 1040 in both MRI and dementia subsample. Participants included in the neuropsychological test and MRI subsamples but excluded from the dementia sample were due to age being greater than to equal to 60 years at exam 7. Participants included in the dementia subsample but not the neuropsychological test or MRI subsamples were due to missing records of neuropsychological testing, or MRI, or both measures within 5 years of exam 7. (TIF) [file pone.0274350.s006.tif]

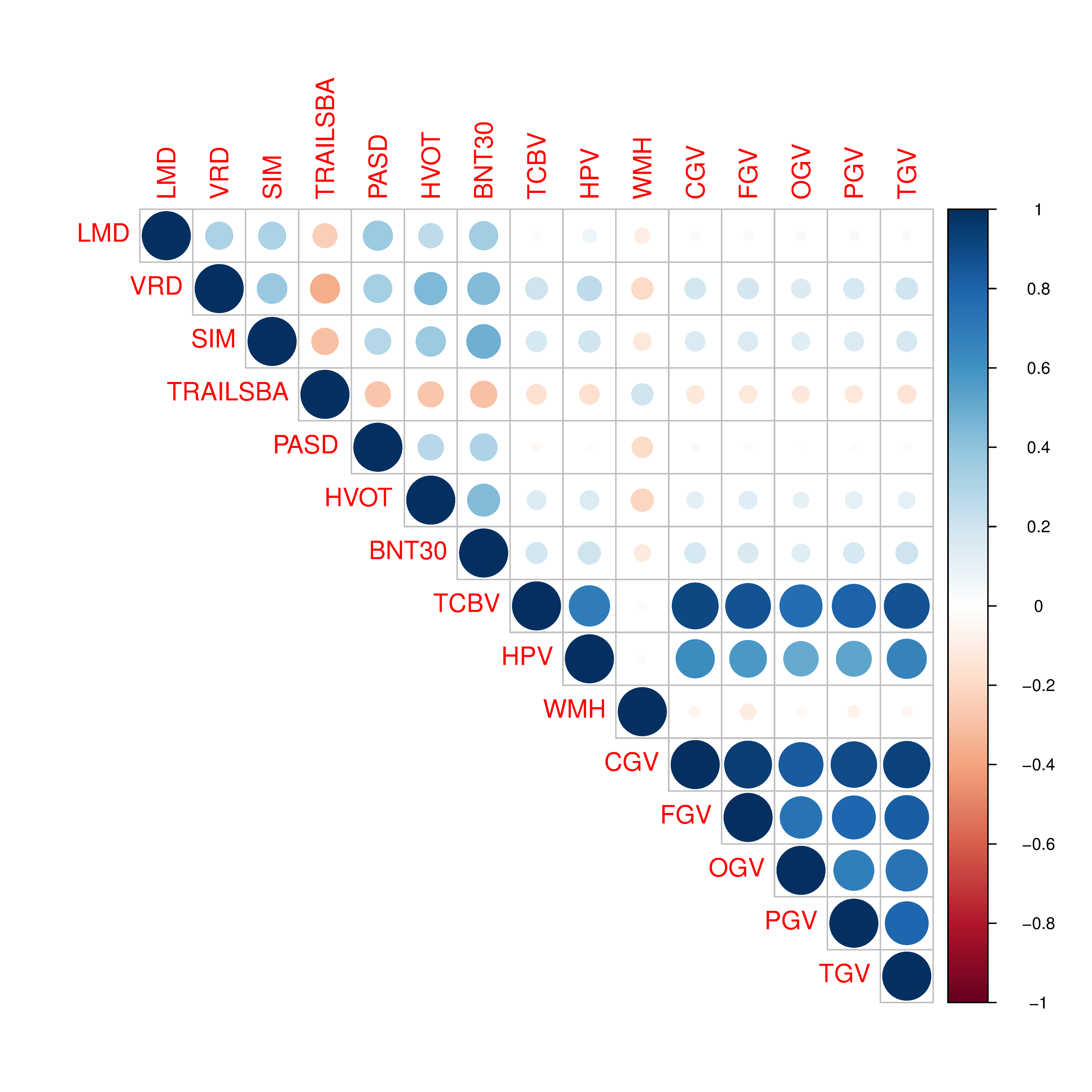

Supplement: S2 Fig — Participants may take cognitive tests and brain MRI measures on different dates. For the 2094 participants included in both the cognitive test and brain MRI samples, there are 2002 participants have these two measures on the same day; among the other 92 participants, 81 have cognitive test prior to the brain MRI measures. The mean difference in dates between the cognitive test and brain MRI measures for those 92 participants is 1.03 years with a standard deviation of 1.23 years. (TIF) [file pone.0274350.s007.tif]

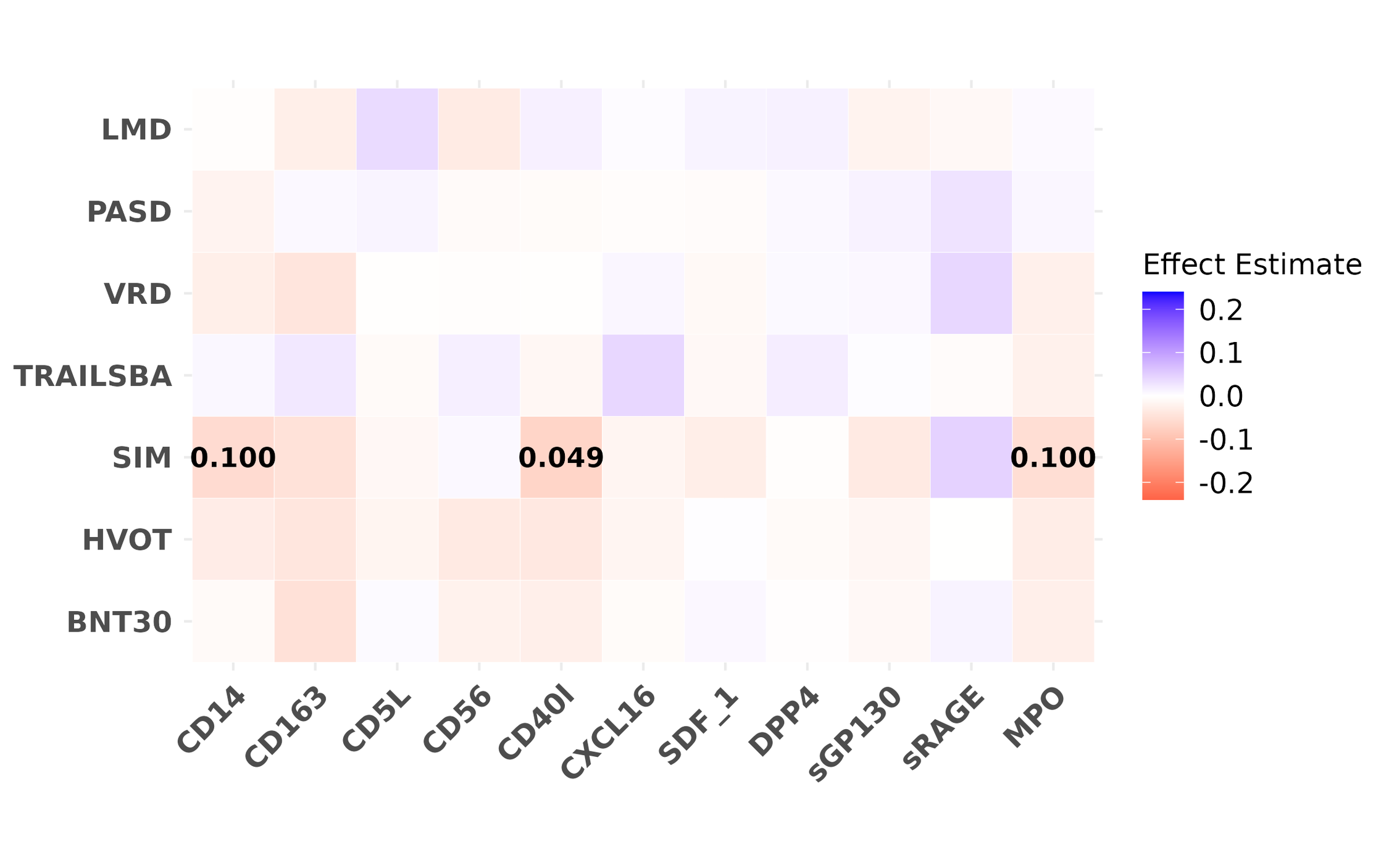

Supplement: S3 Fig — Effect estimates are in colors and FDR value (if ≤0.1) are labeled as numbers. Both protein biomarker predictors and cognitive outcomes were rank normalized to mean 0 and SD 1. Each color block shows the estimated effect for each pair of associations investigated in the primary analyses using linear mixed effect models adjusting for the covariates from Model 2 (Model 1 covariates plus APOE ε4 carrier status and CVD risk factors: SBP, treatment for hypertension, BMI, current smoking status, total cholesterol levels, HDL, presence of diabetes, prevalent AF, and prevalent CVD). FDR are shown for associations where FDR ≤ 0.1. (TIF) [file pone.0274350.s008.tif]

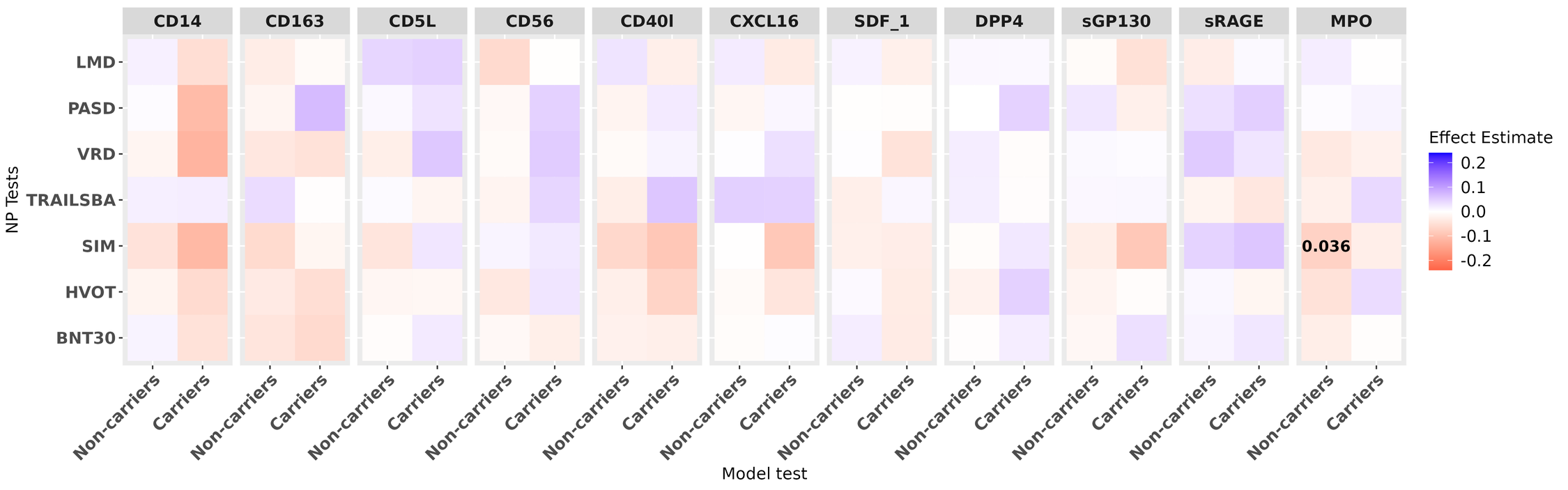

Supplement: S4 Fig — Effect estimates are in colors and FDR value (if ≤0.1) are labeled as numbers. Both protein biomarker predictors and cognitive outcomes were rank normalized to mean 0 and SD 1. Each color block shows the estimated effect for each pair of associations investigated in the primary analyses using linear mixed effect models adjusting for the covariates from Model 1. FDR are shown for associations where FDR ≤ 0.1. (TIF) [file pone.0274350.s009.tif]

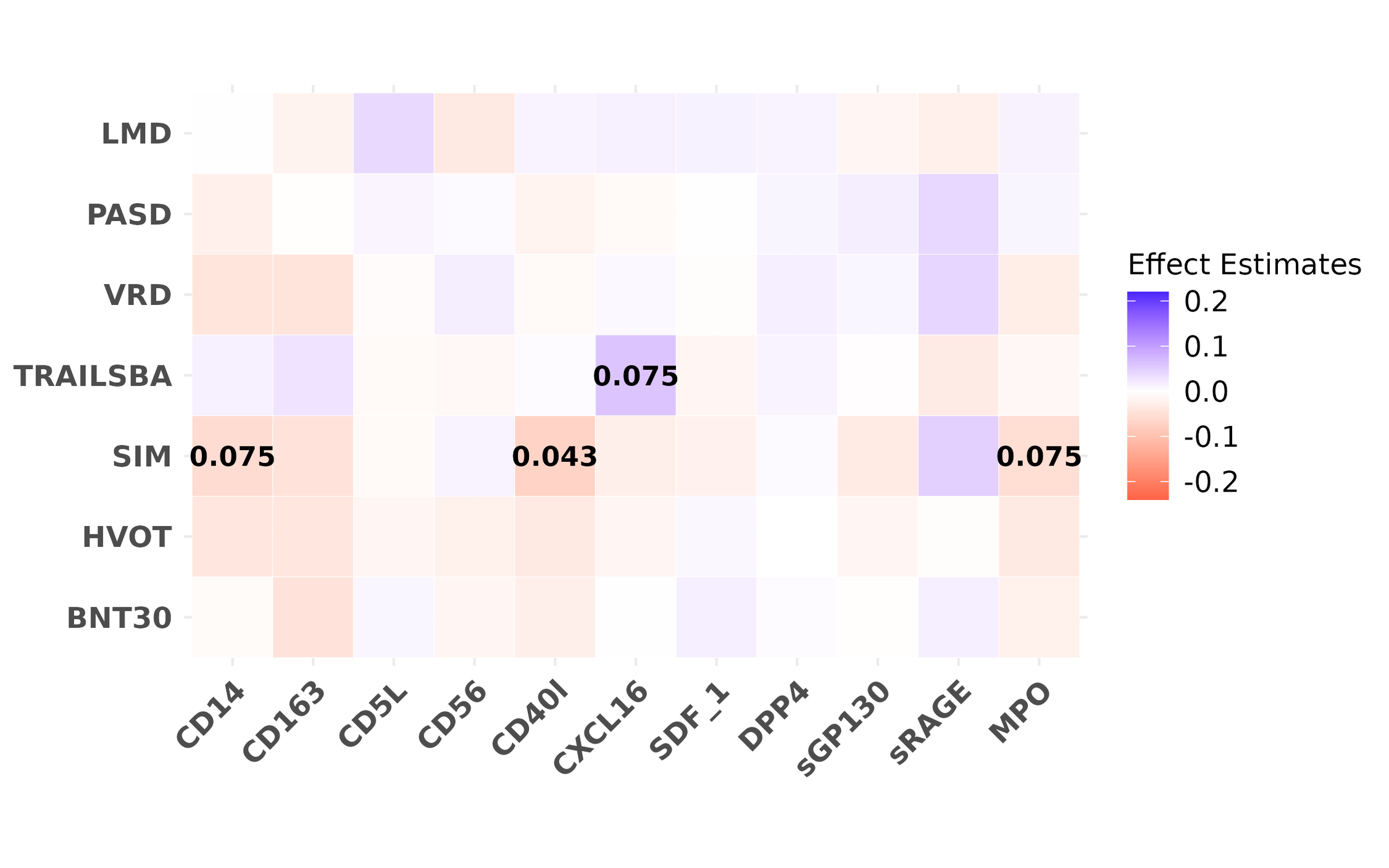

Supplement: S5 Fig — Effect estimates are in colors and FDR value (if ≤0.1) are labeled as numbers. Both protein biomarker predictors and cognitive outcomes were rank normalized to mean 0 and SD 1. Each color block shows the estimated effect for each pair of associations investigated in the primary analyses using linear mixed effect models adjusting for the covariates from Model 2. FDR are shown for associations where FDR ≤ 0.1. (TIF) [file pone.0274350.s010.tif]

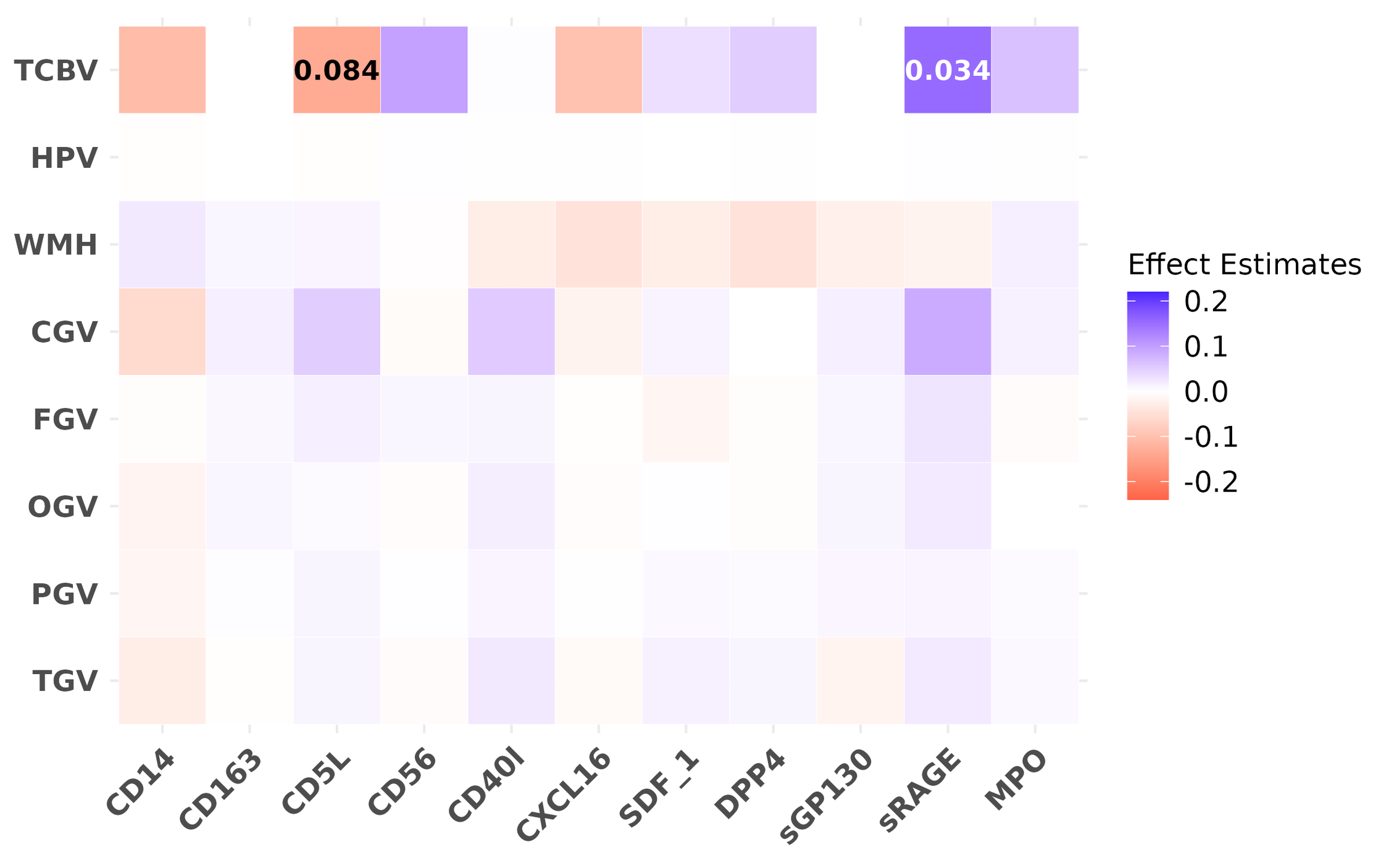

Supplement: S6 Fig — Effect estimates are in colors and FDR value (if ≤0.1) are labeled as numbers. Protein biomarker predictors were rank normalized to mean 0 and SD 1. Total and regional brain volumes and WMH volume were as percentage of total cranial volume, WMH was also log transformed. Each color block shows the estimated effect for each pair of associations investigated in the primary analyses using linear mixed effect models adjusting for the covariates from Model 2 (Model 1 covariates plus APOE ε4 carrier status and CVD risk factors: SBP, treatment for hypertension, BMI, current smoking status, total cholesterol levels, HDL, presence of diabetes, prevalent AF, and prevalent CVD). FDR are shown for associations where FDR ≤ 0.1. (TIF) [file pone.0274350.s011.tif]

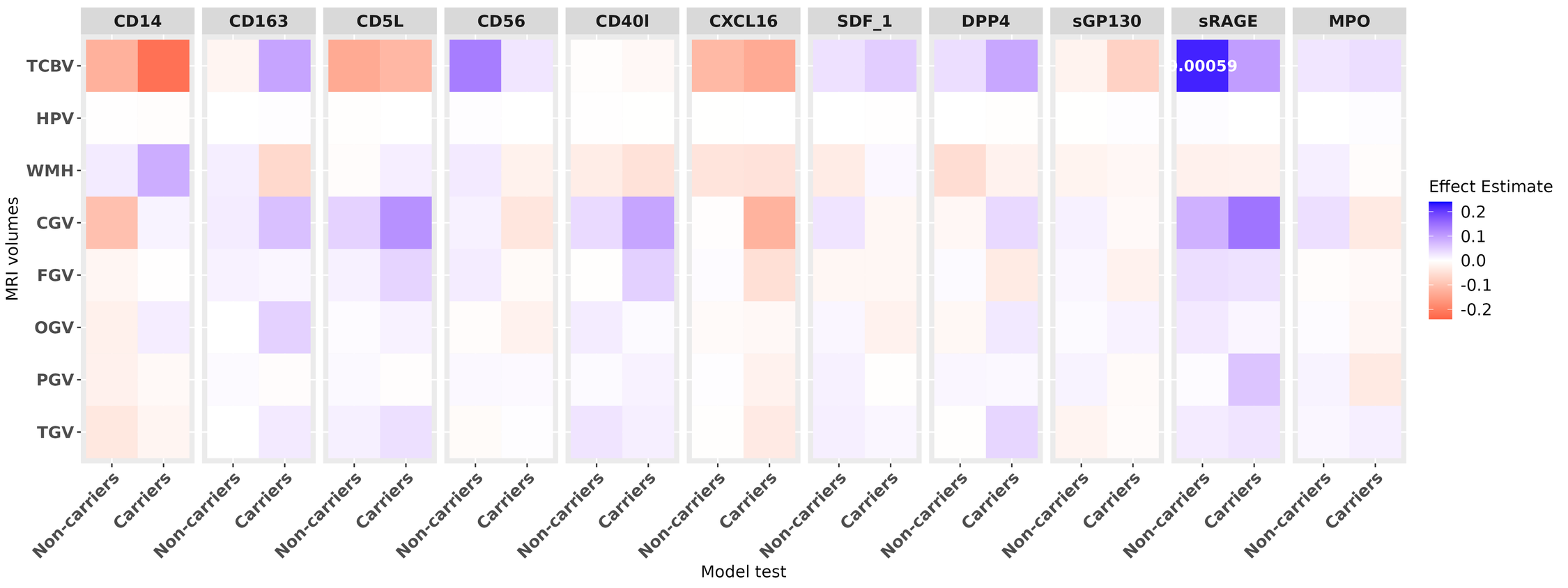

Supplement: S7 Fig — Effect estimates are in colors and FDR value (if ≤0.1) are labeled as numbers. Protein biomarker predictors were rank normalized to mean 0 and SD 1. Total and regional brain volumes and WMH volume were as percentage of total cranial volume, WMH was also log transformed. Each color block shows the estimated effect for each pair of associations investigated in the primary analyses using linear mixed effect models adjusting for the covariates from Model 1. FDR are shown for associations where FDR ≤ 0.1. (TIF) [file pone.0274350.s012.tif]

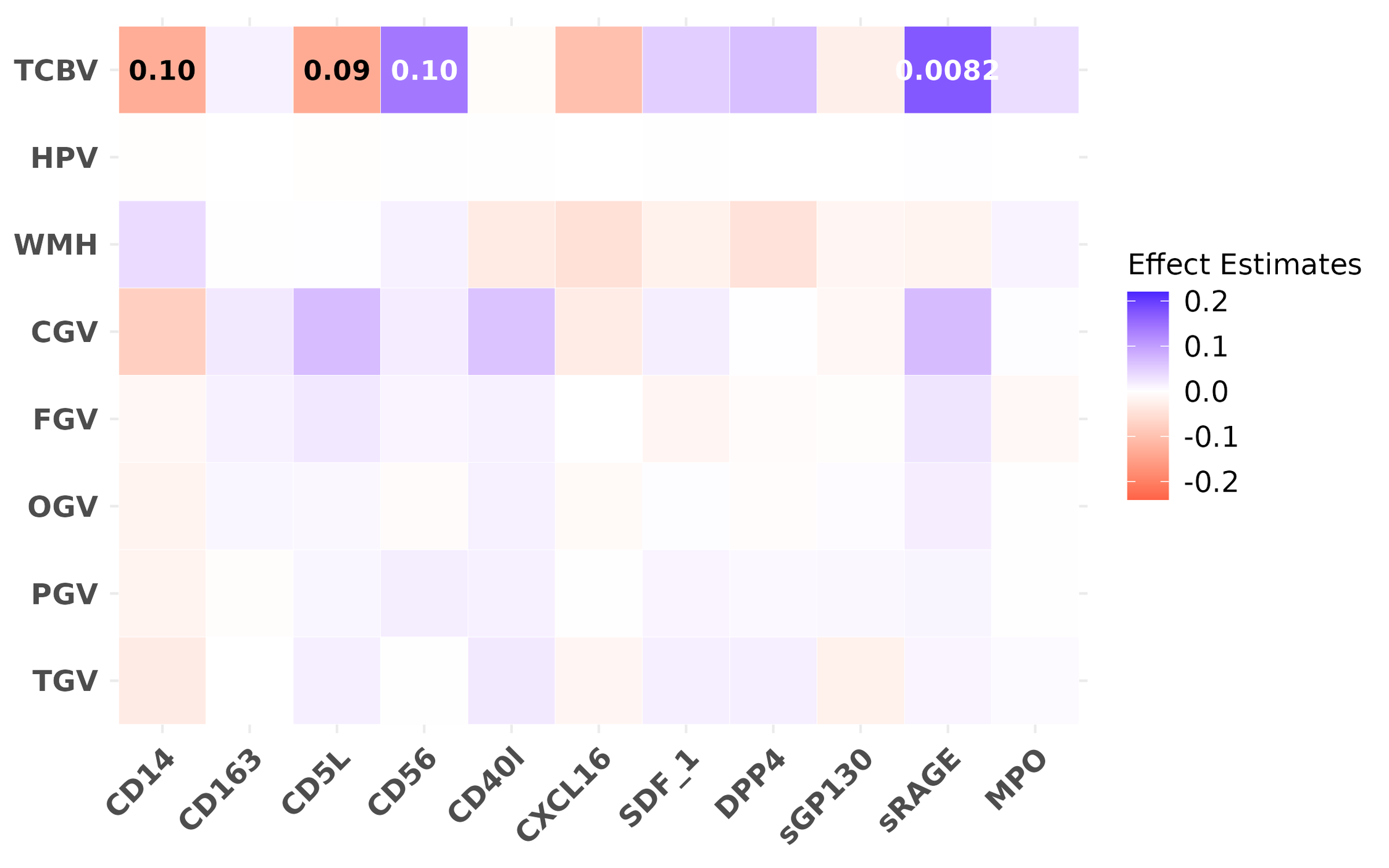

Supplement: S8 Fig — Effect estimates are in colors and FDR value (if ≤0.1) are labeled as numbers. Protein biomarker predictors were rank normalized to mean 0 and SD 1. Total and regional brain volumes and WMH volume were as percentage of total cranial volume, WMH was also log transformed. Each color block shows the estimated effect for each pair of associations investigated in the primary analyses using linear mixed effect models adjusting for the covariates from Model 1. FDR are shown for associations where FDR ≤ 0.1. (TIF) [file pone.0274350.s013.tif]
